# More Light

**Published:** 1866-05

**Authors:** A. Lawrence

**Affiliations:** Lowell, Mass.


### "MORE LIGHT."

BY DR. A. LAWRENCE, LOWELL, MASS.

Permit me to call the attention of the Dental Profession, and the scientific world generally, to the new light on the subject of steam, as set forth in an article in the "Register" for Dec. 1865, p. 505, by a writer, whose modesty deprives the public of all but one initial of his name.

The third paragraph of the article is so lucid, and yet so at variance with the vague notions upon the subject usually entertained by those heretofore considered well versed in such matters, that doubtless a new era in steam is about to be ushered in. The ideas advanced are of the utmost importance to the civilized world, upsetting old theories, and pointing out the sure means of rendering the use of steam entirely safe. There is, seemingly, but one difficulty about the new theory, and that is at the starting point, when steam just begins to form, and is what engineers call saturated, or as the writer has it, "lubricated or moistened."

At this point some little care must doubtless be exercised, especially by the old school, to prevent explosions; as we are now to remember that steam is "of the most force," when weakest. After the critical point is passed, with a full supply of fuel, there is nothing to fear; for as extra heat lessens its force, all that is required is to increase the fire, until eventually, if need be, the water is absolutely frozen in the boiler. A patent could probably be obtained for this new method of producing ice, which it is hoped will not be lost to the fortunate inventor.

The laborious experiments which led to the discovery should be remembered, and a monument of steam would be the tribute of respect a grateful public could bestow.

The new absolute theories applied to his experiments, would account for the thermometer standing at 320°, and the guage at 110lbs, on the idea that one or both instruments were imperfect, or not of the best construction, else improperly applied; also that when the thermometer indicated 340°, and the guage only 30lbs, that the steam was escaping at some point where it did not act upon the water in the coil, *if indeed one was used*, and not from an impossibility to create over 30lbs of steam from "one-third of a tumbler of water" in a common vulcanizer; but the improved solution is that the high heat so lessened the force of the steam that no greater pressure could be obtained.

On the 10th of Jan. ult., my assistant put a flask, with a set of teeth imbeded in plaster, into the vulcanizer, with *no water* except what the plaster contained, and yet the case was vulcanized perfectly in an hour, the guage during that time indicated 85lbs., and even more. The neglect to put water into the vulcanizer was purely accidental; and I had supposed, there being no waste of steam, that very little water was sufficient to generate steam enough to act upon the guage, but the deep researches of those more familiar with such matters, learns me where

> "I run the gauntlet of a file of doubts,
> Each one of which down hurls me to the ground."

In this condition I beseech some one to charitably give us "more light."

For the rest, see "Scientific American" for March 3rd, 1866, p. 145.

POSTSCRIPT.—I submit for the special benefit of knowledge thirsting souls my Patent Steam Catechism :—

1st. How much pressure can be created in a "Buckeye" Vulcanizer with $\frac{1}{3}$ of a tumbler of water at 320°, and how much *less* at 340°?

2nd. What is the ratio of *decrease* in the force of steam passing from 320° to 330°, and what temperature above will produce a vaccum ?

3rd. What is the percentage of water in "well lubricated" steam ?

4th. Are boiler explosions caused by a reduction of heat, and the consequent expansion of "lubricated" steam ?

5th. What is the cubic capacity of a "Buckeye" Vulcanizer in inches, and what $\frac{1}{3}$ of a tumbler; also how much steam will $\frac{1}{3}$ of a tumbler of water produce under a temperature of 340° or 320°?

6th. After a given amount of water has been "expanded to its utmost, or crowded into steam" how much of the aqueous fluid will remain for heating ?

7th. At what temperature is steam "expanded to its utmost."?

